# Cardiac arrest reveals a mobile large left atrial myxoma

**DOI:** 10.1002/ccr3.962

**Published:** 2017-04-17

**Authors:** Emmanouel Papadakis, Ilias Vournas, Meletios A. Kanakis

**Affiliations:** ^1^ Onassis Cardiac Surgery CenterAthensGreece

**Keywords:** Arrest, mitral, myxoma

## Abstract

Left atrial myxoma could be a rare cause of cardiac arrest as this mass could impinge on the mitral orifice causing left ventricular inflow tract obstruction.

## Case Image Description

Question: What is this mass prolapsing through the mitral valve orifice in this echo image?

Answer: A left atrial myxoma causing left ventricular inflow tract obstruction.

Cardiac myxoma is the most common type of primary cardiac tumor, but left atrial myxomas causing syncope are found only as scarce cases in the literature 1, 2.

A 39‐year‐old man complaining for severe palpitations had an appointment for transthoracic echocardiography. A cardiac arrest displayed, immediately, after he was placed on the left lateral decubitus position on the examination bed. Emergency resuscitation was successfully employed. The subsequent emergent transthoracic echocardiogram revealed a large mobile left atrial mass consisted with myxoma that was impinging on the mitral orifice, causing temporary complete obstruction of the mitral orifice.

**Figure 1:**
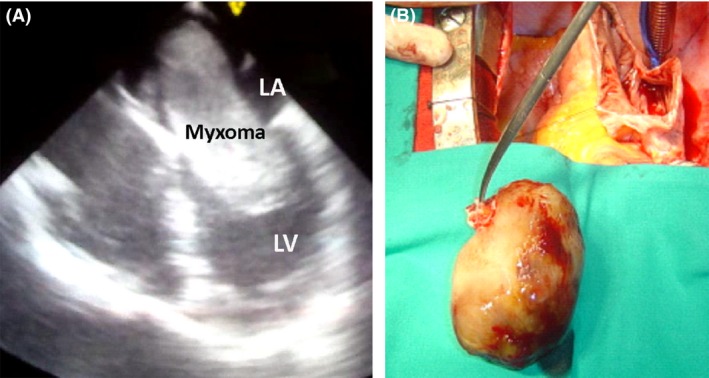
Echocardiography depicting left atrial myxoma causing left ventricular inflow tract obstruction (A). Intraoperative photo showing the excised myxoma (B).

The patient underwent urgent surgical removal of a multilobular mobile mass of 7.2 × 3.5 cm (Fig. 1A and B). Pathology examination confirmed the diagnosis of atrial myxoma. The patient discharged on fifth postoperative day, and he is in an excellent clinical status at 2‐year follow‐up.

## Authorship

EP: conceived of the manuscript idea and participated in its design. IV: helped to draft the manuscript. MAK: participated in the design of the manuscript and drafted the manuscript.

## Conflict of Interest

None declared.

## References

[1] Abernathy, J. H., 3rd , A. B. Locke , and S. K. Shernan . 2006 Dynamic left ventricular inflow obstruction associated with a left atrial myxoma. Anesth. Analg. 103:1406–1407. 17122211 10.1213/01.ane.0000242513.41998.29

[2] Nogueira, D. C. , D. Bontempo , A. C. Menardi , W. V. Vicente , P. J. Ribeiro , and P. R. Evora . 2003 Left atrial myxoma as the cause of syncope in an adolescent. Arq. Bras. Cardiol. 81:206–209, 202–205. 14502389 10.1590/s0066-782x2003001000009

